# Ceramide Aminoethylphosphonate as a New Molecular Target for Pore-Forming Aegerolysin-Based Protein Complexes

**DOI:** 10.3389/fmolb.2022.902706

**Published:** 2022-05-25

**Authors:** Teresa Balbi, Francesco Trenti, Anastasija Panevska, Gregor Bajc, Graziano Guella, Caterina Ciacci, Barbara Canonico, Laura Canesi, Kristina Sepčić

**Affiliations:** ^1^ Department of Earth, Environmental and Life Sciences, University of Genoa, Genoa, Italy; ^2^ Bioorganic Chemistry Laboratory, Department of Physics, University of Trento, Trento, Italy; ^3^ Department of Biology, Biotechnical Faculty, University of Ljubljana, Ljubljana, Slovenia; ^4^ Department of Biomolecular Sciences, University of Urbino Carlo Bo, Urbino, Italy

**Keywords:** aegerolysins, bioinsecticides, ceramide aminoethylphosphonate, hemocytes, marine bivalves, *Mytilus galloprovincialis*, *pleurotus*, toxicity

## Abstract

Ostreolysin A6 (OlyA6) is a 15 kDa protein produced by the oyster mushroom (*Pleurotus ostreatus*). It belongs to the aegerolysin family of proteins and binds with high affinity to the insect-specific membrane sphingolipid, ceramide phosphoethanolamine (CPE). In concert with its partnering protein with the membrane-attack-complex/perforin domain, pleurotolysin B (PlyB), OlyA6 can form bicomponent 13-meric transmembrane pores in artificial and biological membranes containing the aegerolysin lipid receptor, CPE. This pore formation is the main underlying molecular mechanism of potent and selective insecticidal activity of OlyA6/PlyB complexes against two economically important coleopteran plant pests: the western corn rootworm and the Colorado potato beetle. In contrast to insects, the main sphingolipid in cell membranes of marine invertebrates (i.e., molluscs and cnidarians) is ceramide aminoethylphosphonate (CAEP), a CPE analogue built on a phosphono rather than the usual phosphate group in its polar head. Our targeted lipidomic analyses of the immune cells (hemocytes) of the marine bivalve, the mussel *Mytilus galloprovincialis,* confirmed the presence of 29.0 mol% CAEP followed by 36.4 mol% of phosphatidylcholine and 34.6 mol% of phosphatidylethanolamine. Further experiments showed the potent binding of OlyA6 to artificial lipid vesicles supplemented with mussel CAEP, and strong lysis of these vesicles by the OlyA6/PlyB mixture. In *Mytilus* haemocytes, short term exposure (max. 1 h) to the OlyA6/PlyB mixture induced lysosomal membrane destabilization, decreased phagocytic activity, increased Annexin V binding and oxyradical production, and decreased levels of reduced glutathione, indicating rapid damage of endo-lysosomal and plasma membranes and oxidative stress. Our data suggest CAEP as a novel high-affinity receptor for OlyA6 and a target for cytolytic OlyA6/PlyB complexes.

## 1 Introduction

Aegerolysins (Pfam 06355; InterPro IPR009413) are small (13–20 kDa) acidic proteins that have been identified in several eukaryotes and prokaryotes, and are especially abundant in bacteria and mushrooms (Novak et al., 2015; Butala et al., 2017). Several edible oyster mushrooms (e.g., *Pleurotus ostreatus, P. eryngii, P. pulmonarius*) harbour various highly identical (78%–98%) aegerolysin sequences in their genomes (Panevska et al., 2021). The most prominent feature of these *Pleurotus* aegerolysins is their ability to specifically interact with selected membrane lipids and lipid domains (Butala et al., 2017; Grundner et al., 2021; Panevska et al., 2021). Furthermore, *Pleurotus* aegerolysins can act in concert with pleurotolysin B (PlyB) or erylysin B, highly (>95%) identical 59-kDa protein partners that have a membrane-attack-complex/perforin (MACPF) domain and are produced by *P. ostreatus* and *P. eryngii,* respectively (Tomita et al., 2004; Ota et al., 2013; Lukoyanova et al., 2015; Milijaš Jotić et al., 2021). Upon binding to the membrane lipid receptor, aegerolysin recruits the MACPF-partnering protein that undergoes extensive conformational changes and penetrates the membrane. The final bi-component transmembrane pore is composed of 13 MACPF-protein molecules, each sitting atop of an aegerolysin dimer (Ota et al., 2013; Lukoyanova et al., 2015; Milijaš Jotić et al., 2021). This pore formation results in direct cell death, or in the creation of a passageway for other molecules that can kill the cell.

In particular, the aegerolysin ostreolysin A6 (OlyA6) from the edible oyster mushroom (*P. ostreatus*) can interact with moderate affinity (*k*_*D*_∼ 1 μM) with membrane nanodomains enriched in sphingomyelin and cholesterol (Chol) (Skočaj et al., 2014; Bhat et al., 2015), where it specifically senses the Chol-bound conformation of sphingomyelin (Endapally et al., 2019), and can therefore be applied as ideal non-toxic marker for visualization of the structure and dynamics of membrane rafts in living mammalian cells (Skočaj et al., 2014; Endapally et al., 2019). Furthermore, OlyA6 can interact with high affinity (*k*_*D*_∼ 1 nM) with artificial lipid vesicles and biological membranes that contain physiologically relevant concentrations (1–5 mol%) of ceramide phosphoethanolamine (CPE) (Bhat et al., 2015; Panevska et al., 2019a; Novak et al., 2020; Panevska et al., 2021), which is the major sphingolipid in invertebrate cell membranes and is not found in other taxa (Panevska et al., 2019b). Through this CPE-binding, the OlyA6/PlyB cytolytic complexes have been shown to act as potent and species-specific bioinsecticides, for use against selected coleopteran pests, such as western corn rootworm and Colorado potato beetle (Panevska et al., 2019a). We confirmed that the molecular mechanism of action of these insecticidal protein complexes arises from their specific interactions with their membrane lipid receptor, the CPE (Milijaš Jotić et al., 2021) at the acidic conditions that are characteristic for beetles’ midgut.

**FIGURE 1:**
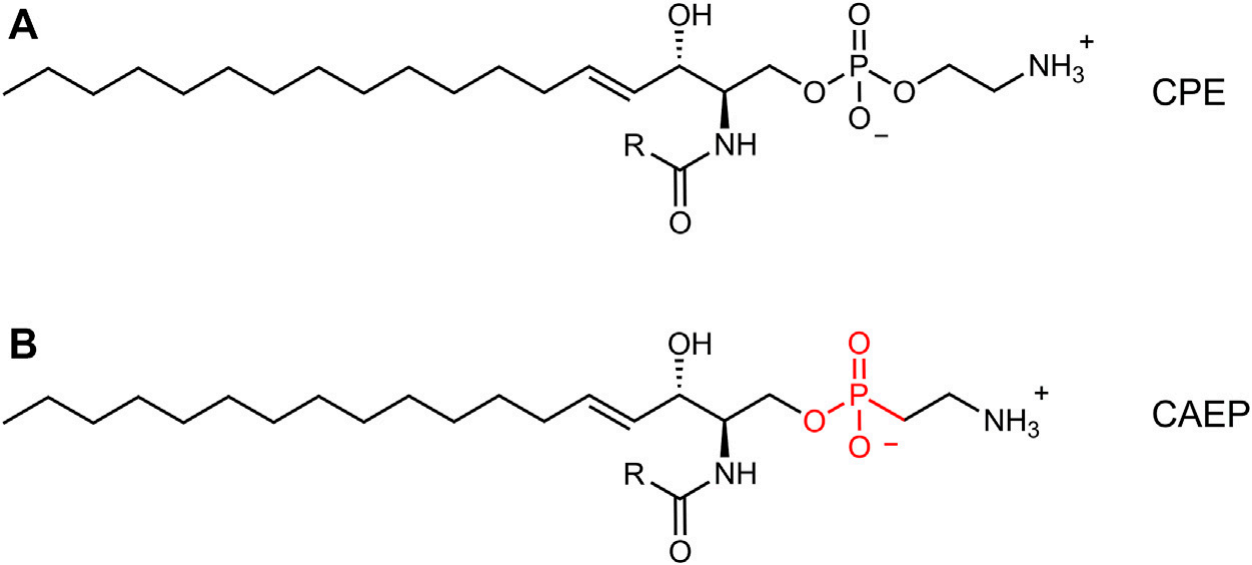
Comparison between canonical ceramide phosphoethanolamine (CPE, **(A)** and ceramide aminoethylphosphonate (CAEP, **(B)**. The latter bears a phosphonate headgroup instead of a phosphate group.

In addition to arthropods (Crone and Bridges, 1963; Masood et al., 2010; Kraut, 2011; Panevska et al., 2019a), the presence of CPE was also found in deep-sea mussels (Kellermann et al., 2012) and some marine gastropods (sea snails) (Hori et al., 1967), protozoa (Broad and Dawson, 1973; Kaneshiro et al., 1997), oomycetes (Moreau et al., 1998), and Bacteroidetes (Batrakov et al., 2000). Moreover, marine invertebrates (bivalves, gastropods, cephalopods, oysters, sea anemones, hydrocorals) synthesize as the main membrane component an even more peculiar sphingophosphonolipid, ceramide-2-aminoethylphosphonate (CAEP), which contains a carbon-phosphorus bond and has a unique triene type of sphingoid base in its structure (Moschidis, 1984; Tomonaga et al., 2017; Imbs et al., 2019). Also, some marine protozoa, bacteria (*Bdellovibrio bacteriovous*), and plant pathogen oomycetes (*Pythium prolatrum*) can contain CAEP in their membranes (Moschidis, 1984), but in mammals this lipid is very rare, as it is the CPE. The CAEP lipid has 2-aminoethylphosphonate as its polar head group, with a phosphorus atom directly attached to a carbon atom (C-P bond) (Tomonaga et al., 2017), in contrast to the C-O-P bond found in phosphoethanolamine as the polar head group of CPE (Figure 1). The data on the physical properties and morphology of CAEP-containing membranes are still sparse in the literature and have not been studied in detail. Although there is a lack of information on the physical properties of this lipid, CAEP has a high structural similarity to CPE, and is considered as even more stable molecule than CPE (Vacaru et al., 2013). The biological role of CAEP has not been fully elucidated, but it is thought to have similar activity in marine invertebrates as the major mammalian sphingolipid, sphingomyelin (Wang et al., 2020).

Recent analysis of the lipidome of the marine bivalve, the Mediterranean mussel (*Mytilus galloprovincialis*) revealed the presence of CAEP species (Donato et al., 2018). During our evaluation of toxicity of insecticidal complexes based on *Pleurotus* aegerolysins on various target and non-target terrestrial and aquatic invertebrates, we explored the possibility that these protein complexes, in particular OlyA6/PlyB, might exert toxicity also against mussel cells. In this regard, mussel immune cells (hemocytes), a widespread experimental model to evaluate the effects and mechanisms of action of different chemicals in mussels (Katsumiti et al., 2019; Balbi et al., 2021), were utilized as a model to further explore the lipid composition of *Mytilus* cells, to identify the possible OlyA6 membrane lipid receptor, and to evaluate the toxicity of OlyA6/PlyB complexes.

## 2 Results

### 2.1 Lipidome Analysis and CAEP Isolation From *M. galloprovincialis* Hemocytes

**FIGURE 2:**
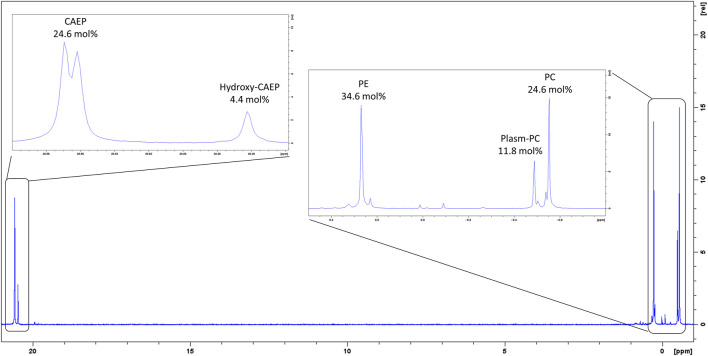
^31^P-NMR spectrum of *M. galloprovincialis* hemocytes raw extract.

**FIGURE 3:**
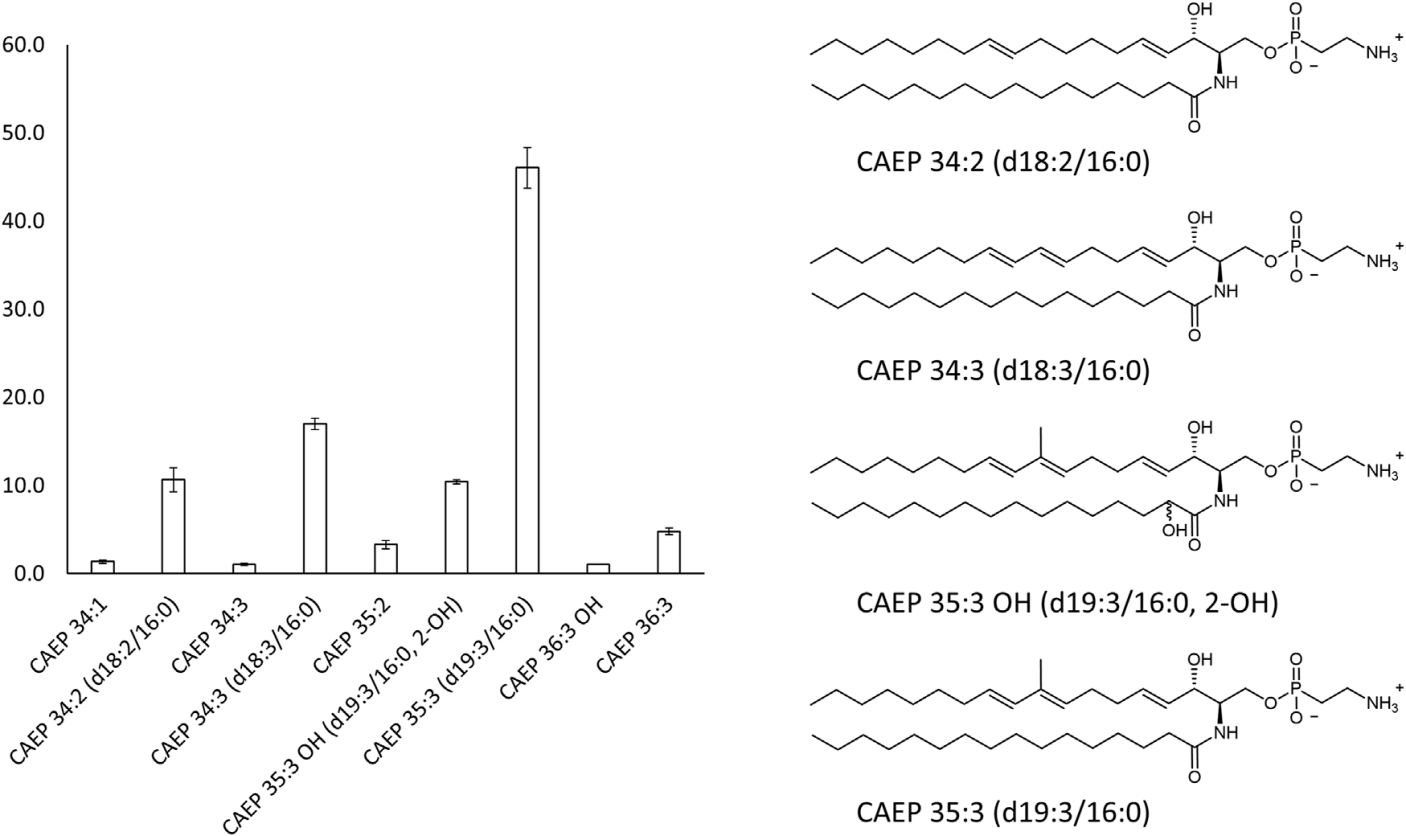
Main CAEP relative components of the *M. galloprovincialis* hemocytes raw extract (>3 mol%) and their corresponding structures.

Our ^31^P-NMR analyses of lipids extracted from *M. galloprovincialis* hemocytes showed three major phospholipids classes: phosphatidylcholine (PC); phosphatidylethanolamine (PE) and CAEP. The latter represented 29.0 mol% of all phospholipids, with a contribution of 4.4 mol% hydroxylated CAEP and 24.6 mol% miscellaneous CAEP counting different chain length and degree of unsaturation. PC and PE contributed to the phospholipid content by 36.4 mol% and 34.6 mol%, respectively (Figure 2). Phosphatidylcholines were found as a mixture of canonical PC and plasmenyl-PC, accounting for the 24.6 mol% and 11.8 mol%, respectively. ^1^H-NMR spectrum allowed us to estimate the membrane fluidity by calculating the ratio between PC and total Chol. We found the value of PC/Chol ratio to be 1.6, indicating a presence of about 22 mol% of Chol in mussel hemocytes. A thorough analysis of the ^1^H and ^13^C-NMR spectra (including 2D measurements) of the raw extract allowed to establish the structural features of the isolated CAEP lipids as summarized in Figure 3.

In particular, the ethylamino moieties of PE and CAEP were clearly identified by the ^1^H-NMR signal (Supplementary Figure S1). In fact, the methylene group–**CH**_**2**_-NH_2_ in CAEP is characterized by geminal protons at δ_H_ 3.09 td (7.0, 13.9 Hz) coupled to δ_C_ 36.9 whilst the corresponding methylene group of PE shows signals at δ_H_ 3.13 brt (7.0 Hz) coupled to δ_C_ 41.9 (Supplementary Figure S1). The coupling pattern of the former resulting from the significant ^3^J (P,H) value (13.9 Hz) is expected only in CAEP lipids. Differences were even more significant on the adjacent methylene group (–**CH**_**2**_-CH_2_NH_2_) whose resonances in CAEP are strongly shielded (δ_H_ 1.87 td (7.2, 15.9 Hz); δ_C_ 32.7) with respect to PE (δ_H_ 4.03 t (7.0); δ_C_ 67.3). As outlined below, the most abundant CAEP found in the extract showed a strong UV absorption at λ 232 nm, suggesting the presence of a conjugated diene moiety in the sphingosine backbone, beside the expected isolated C (4) = C (5) double bond. We suggest here that in our CAEP we are dealing with a (8*E*,10*E*) diene system where the signals for the “inner” (H-9 and H-10) and the “outer” olefinic protons (H-8 and H-11) coalesced giving two signals at δ_H_ 6.02 d (15.3 Hz) and δ_H_ 5.53 dt (15.3, 7.2 Hz), respectively. The analysis of the coupling patterns, further supported by 2D-NMR spectra (HSQC, HMBC and COSY) allowed us to establish that the sphingosine moiety of these CAEP is built on a (4E, 8E, 10E) triene system. It is worth noting that several CAEP lipid species contain a sphingosine backbone with an odd number of carbon atoms (19); our NMR data strongly suggest that in these species the C (9) carbon atom of the conjugated diene system is methylated (δ_H_ 1.71 (s)). Concerning the CAEP bearing an extra–OH group, NMR analysis indicated that it resides on the C (2’) of the fatty acyl chain (δ_H_ 3.99 m; δ_C_ 72.7).

The overall profile of CAEP in term of sphingosine/amide chain lengths and unsaturation was then established by liquid chromatography mass-spectrometry (LC-MS) and LC-MS-MS measurements. The most abundant CAEP species present in the raw extract were (Figure 3): CAEP 35:3 OH ((= CAEP d19:3/16:0, 2-OH), 10 mol%); CAEP 34:3 ((= CAEP d18:3/16:0), 18 mol%); CAEP 35:3 ((= CAEP d19:3/16:0), 47 mol%) and CAEP 34:2 ((= CAEP d18:2/16:0), 11 mol%). Minor CAEP lipids, including species bearing an N-methyl-amino, were also found, but their abundance did not exceed 3 mol%.

The components of the CAEP bearing the conjugated diene system on the sphingosine backbone were isolated from the raw extract by high pressure liquid chromatography (HPLC-UV (*λ* = 234 nm) chromatography) (Supplementary Figure S2) and they were then tested as a mixture in affinity bioassays.

### 2.2 OlyA6/PlyB Interaction With Artificial Lipid Membranes Containing Mussel CAEP

**FIGURE 4:**
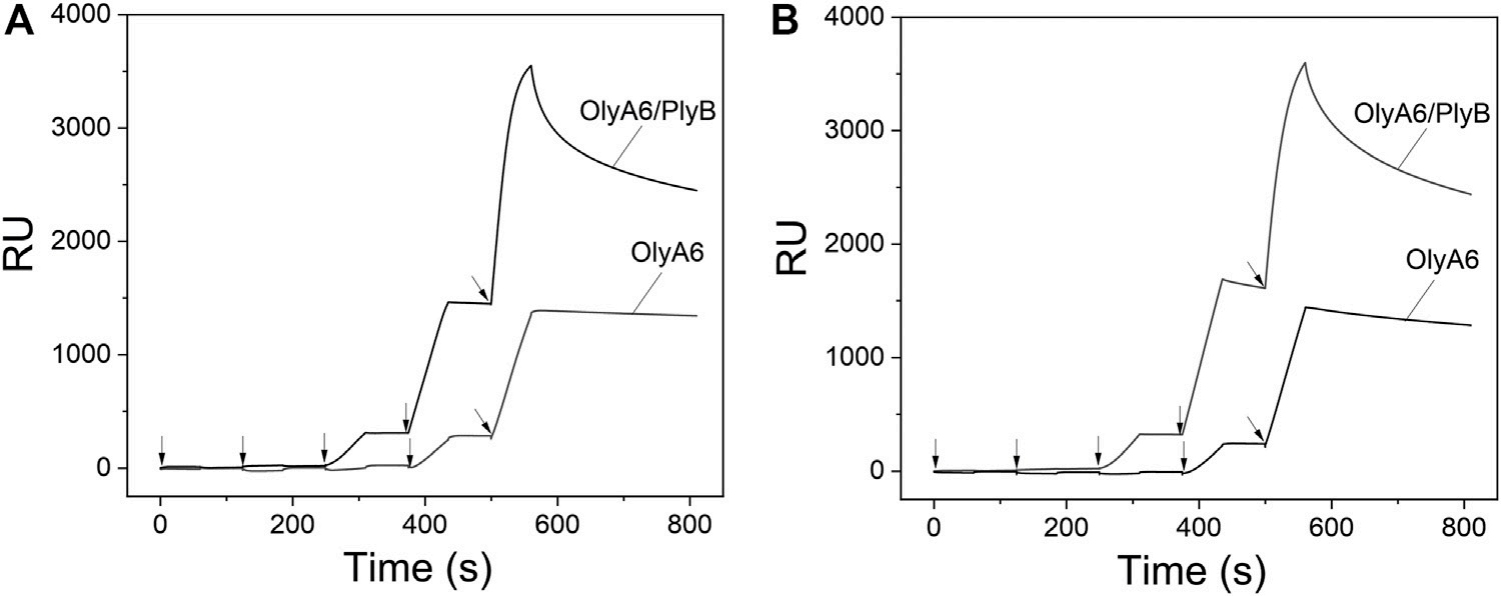
Binding of OlyA6 and OlyA6/PlyB (12.5/1, molar ratio) to immobilized equimolar CAEP/POPC/Chol **(A)** and CPE/POPC/Chol **(B)** large unilamellar vesicles using the kinetic titration approach in a single cycle by successive injections of 0.03, 0.06, 0.12, 0,25, and 0.5 μM (from left to right) concentration. Vesicles were immobilized on the Biacore L1 chip to approximately 8,000 response units (RU). Representative sensorgrams from two independent experiments are shown. CPE-ceramide phosphoethanolamine, Chol-cholesterol, POPC-1-palmitoyl-2-oleoyl-*sn*-glycero-3-phosphocholine, CAEP-ceramide aminoethylphosphonate.

Surface plasmon resonance studies with large unilamellar vesicles (LUVs) immobilized on a chip revealed the interaction of the *Pleurotus* aegerolysin OlyA6 with artificial lipid membranes composed of an equimolar mixture of CAEP (isolated from mussel hemocytes), palmitoyl-oleoyl-phosphatidylcholine (POPC), and Chol. We applied a kinetic titration approach by injecting OlyA6 at five concentrations ranging from 0.03 to 0.5 μM over the LUVs immobilized on the chip, without dissociation time between protein injections. The resulting sensorgrams showed that OlyA6 interacted strongly and irreversibly with CAEP-enriched membranes (Figure 4A). The interaction of OlyA6 with CAEP-enriched LUVs was considerably stronger in the presence of PlyB (Figure 4A), indicating the formation of more stable proteolipid complexes. The binding kinetics of OlyA6 to CAEP-containing membranes was comparable with the interaction of OlyA6 with CPE-containing LUVs (Figure 4B), and in both cases the dissociation phase indicated an irreversible interaction with these lipid membranes, alone or in the presence of PlyB.

**FIGURE 5:**
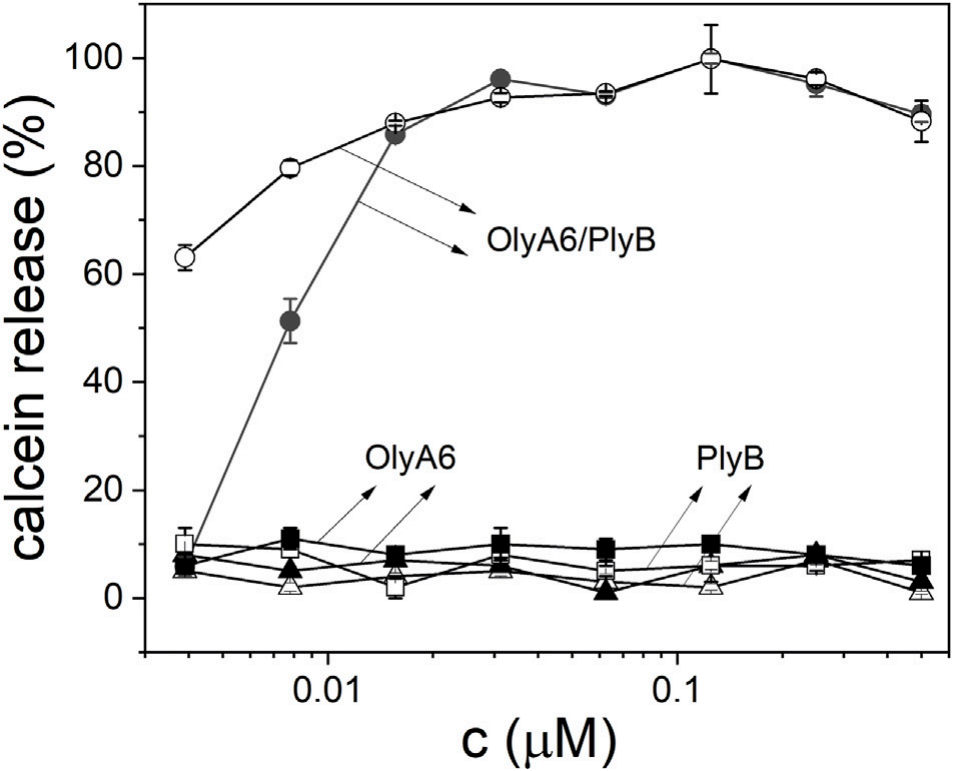
Concentration dependence of permeabilization of equimolar CAEP/POPC/Chol (full symbols) and CPE/POPC/Chol (open symbols) small unilamellar vesicles by OlyA6/PlyB. OlyA6/PlyB molar ratio = 12.5/1. Individual proteins, OlyA6 and PlyB, were also tested as controls. CPE-ceramide phosphoethanolamine, Chol-cholesterol, POPC-1-palmitoyl-2-oleoyl-*sn*-glycero-3-phosphocholine, CAEP-ceramide aminoethylphosphonate.

Monitoring of the fluorescence of calcein released from the small unilamellar equimolar lipid vesicles composed of CAEP/POPC/Chol confirmed the concentration-dependent membrane permeabilization by OlyA6 in combination with PlyB (Figure 5). As in the binding studies using surface plasmon resonance, the lytic activity of OlyA6/PlyB on equimolar CPE/POPC/Chol membranes was comparable to the lytic activity of OlyA6/PlyB on the newly studied CAEP-containing membranes. However, the OlyA6/PlyB protein complex showed slightly higher permeabilization on CAEP-containing membranes compared with CPE-containing membranes when tested at lower (<15 nM) protein concentrations (Figure 5). OlyA6 and PlyB alone did not induce vesicle permeabilization (Figure 5).

### 2.3 Effect of Aegerolysin-Based Complexes on Haemocytes of *Mytilus galloprovincialis*

**FIGURE 6:**
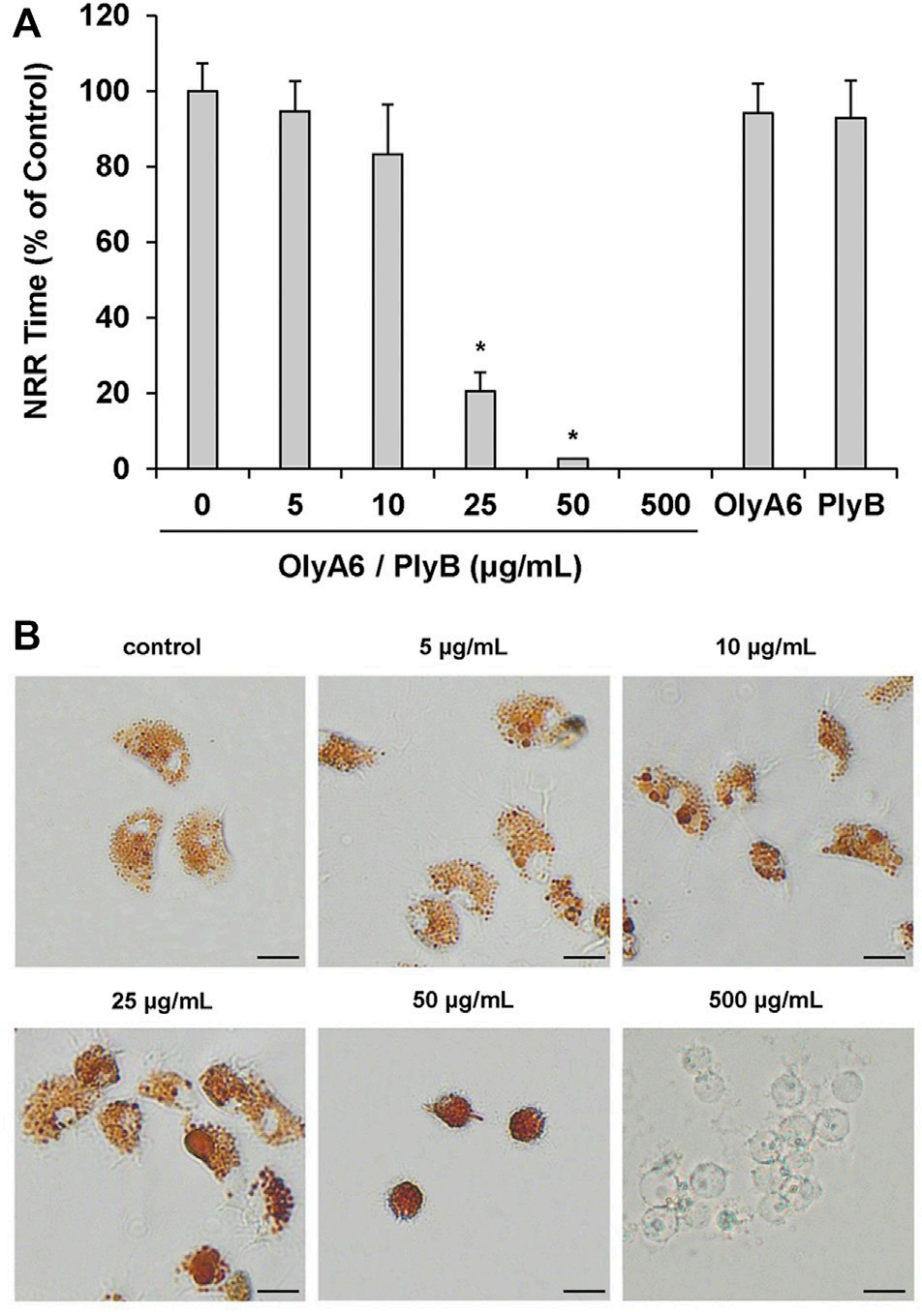
Effects of the OlyA6/PlyB mixture on lysosomal membrane stability of *M. galloprovincialis* hemocytes. Hemocytes were exposed to different concentrations of the mixture (5, 10, 25, 50, 500 μg/ml; OlyA6/PlyB molar ratio 12.5/1) and to OlyA6 (25 μg/ml) or PlyB alone (2 μg/ml). **(A)** Data, reported as percent values of neutral red retention time with respect to controls, representing the mean ± SD of four experiments in triplicate, were analysed by the Mann Whitney *U* test. *p < 0.01. **(B)** Representative images of hemocytes treated with different concentrations of the protein mixture, as indicated. NRR–neutral red retention time. Scale bar = 10 μm.

**FIGURE 7:**
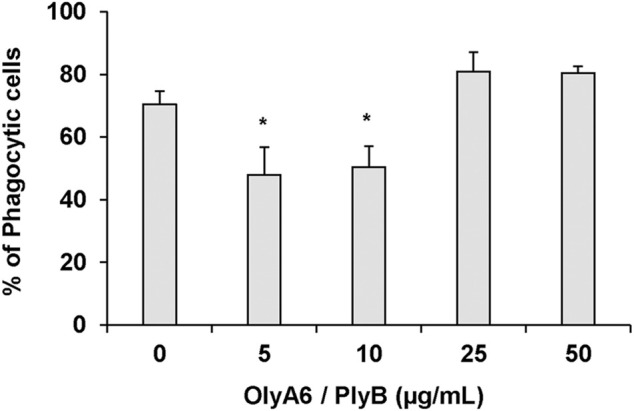
Effects of the OlyA6/PlyB mixture on phagocytic activity of *M. galloprovincialis* hemocytes. Hemocytes were exposed to different concentrations of the protein mixture (5, 10, 25, 50 μg/ml; OlyA6/PlyB molar ratio 12.5/1). Data, representing the mean ± SD of four experiments in triplicate, were analysed by the Mann Whitney *U* test. *p < 0.01.

In *Mytilus* hemocytes exposed to the OlyA6/PlyB mixture for 30 min, lysosomal membrane stability was evaluated as a marker of cellular stress by the neutral red retention (NRR) time assay, and the results are shown in Figure 6. OlyA6/PlyB induced a dose-dependent decrease in hemocyte lysosomal membrane stability, with an EC_50_ of 6.5 μg/ml (95% CI: 2.305–18.2), whereas OlyA6 and PlyB alone were ineffective (Figure 6A). Optical microscopy observations for each concentration tested are also reported (Figure 6B). Lower concentrations of the protein mixture (5 and 10 μg/ml) did not affect hemocyte lysosomal membrane stability, whereas a significant decrease was observed at 25 μg/ml (−80%; *p* < 0.01). At this concentration, several cells with red cytosol, indicating neutral red leakage from lysosomes, and round shaped, due to detachment from the substrate, were observed (Figure 6B). At 50 μg/ml, lysosomal membranes were completely destabilized, and all the cells were rounded, indicating cell detachment and death (−98%; *p* < 0.01) (Figure 6B). Interestingly, the highest concentration tested (500 μg/ml) apparently caused complete loss of cytoplasm and organelles, and only cell membranes (“ghost” hemocytes) could be observed (Figure 6B). Exposure of mussel hemocytes to different concentrations of the OlyA6/PlyB mixture for 30 min also induced a significant decrease in the percentage of phagocytic cells with respect to controls at 5 and 10 μg/ml (about −30% for both concentrations; *p* < 0.01), while higher concentrations were ineffective (Figure 7).

**FIGURE 8:**
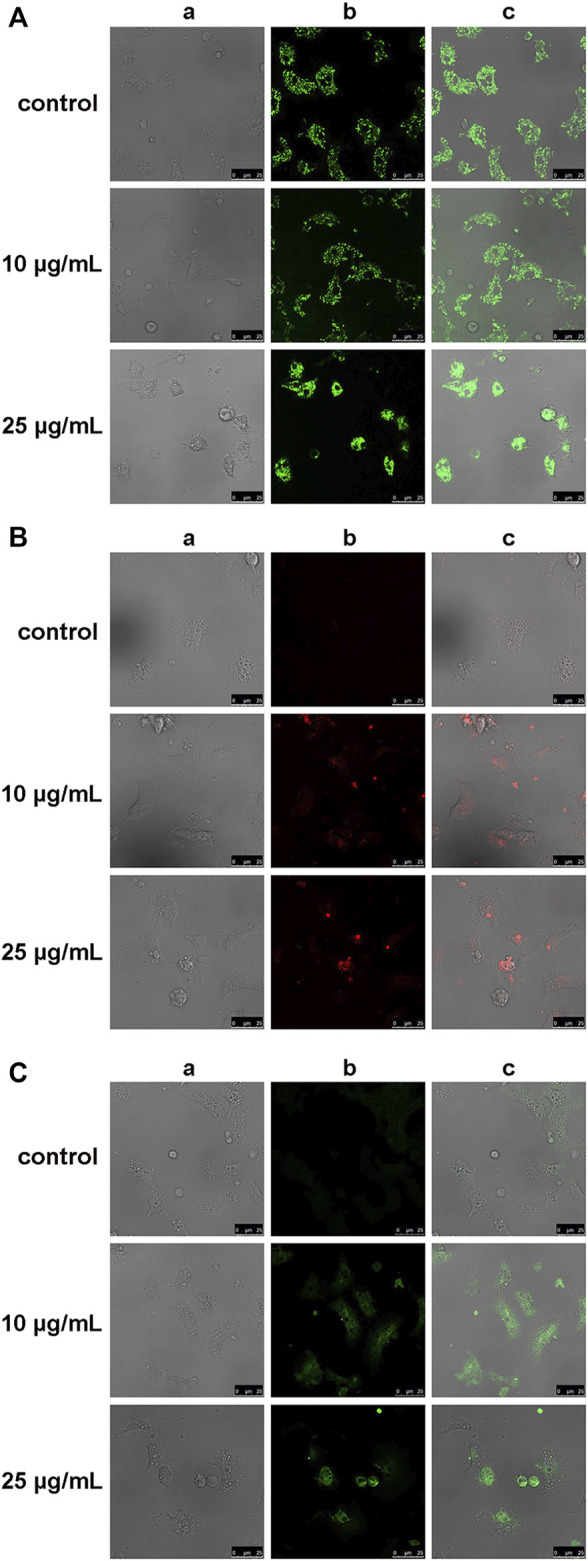
Effects of the OlyA6/PlyB mixture on mitochondrial and oxidative stress parameters in *M. galloprovincialis* hemocytes evaluated by confocal laser scanning microscopy. Representative images of hemocytes exposed to different concentrations of the OlyA6/PlyB mixture at the OlyA6/PlyB molar ratio 12.5/1 and loaded with **(A)** tetramethylrhodamine ethyl ester (TMRE), for mitochondrial membrane potential; **(B)** MitoSOX Red for mitochondrial superoxide (O_2_^−^) production; **(C)** CM-H2DCFDA (C-DCF), for generation of intracellular H_2_O_2_. Rows correspond to different concentrations of the OlyA6/PlyB mixture (0, 10 and 25 μg/ml). Columns show: **(a)** brightfield, **(b)** fluorescence signals of TMRE, MitoSOX and C-DCF **(c)** merged channels. Scale bar: 25 μm.

The effects of hemocyte incubation with OlyA6/PlyB (10 and 25 μg/ml) on mitochondrial and oxidative stress parameters were evaluated by cell staining with TMRE, MitoSOX and C-DCF, for determination of mitochondrial membrane potential, mitochondrial superoxide (O_2_^**.**-^) and intracellular hydrogen peroxide (H_2_O_2_) production, respectively. Representative confocal laser scanning microscopy images are reported in Figure 8. At the lowest concentration tested (10 μg/ml), OlyA6/PlyB exposure did not affect the TMRE signal and cell morphology, whereas at 25 μg/ml the cells were rounded and showed a clear increase in fluorescence with respect to controls, indicating decreased mitochondrial membrane potential (Figure 8A). The MitoSOX and C-DCF signals were barely detectable in control hemocytes, but a progressive increase was observed in cells exposed to both concentrations of the OlyA6/PlyB mixture with respect to controls (Figures 8B,C), demonstrating the stimulation of reactive oxygen species production.

**FIGURE 9:**
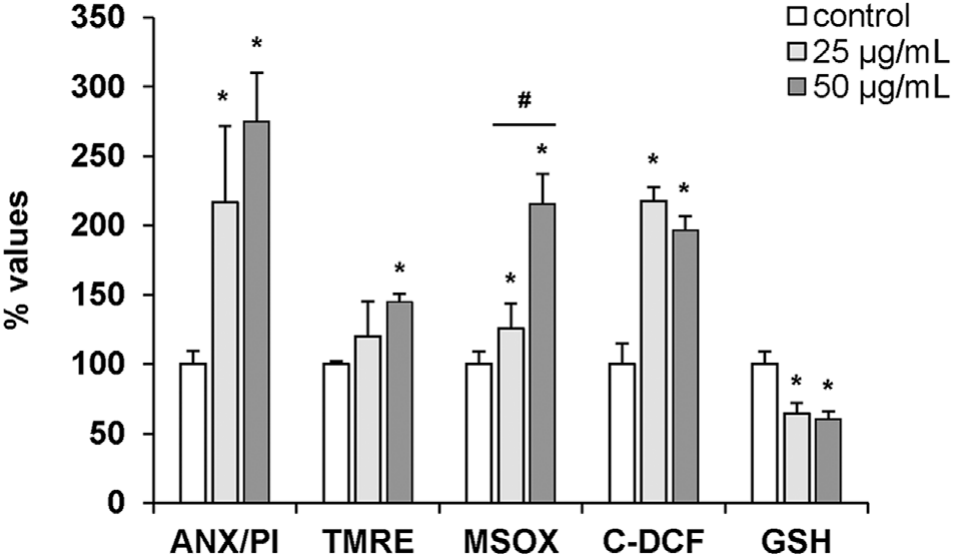
Effects of the OlyA6/PlyB mixture on apoptotic and oxidative stress parameters in *M. galloprovincialis* hemocytes evaluated by flow cytometry. Hemocytes were exposed to the OlyA6/PlyB mixture (25 and 50 μg/ml; OlyA6/PlyB molar ratio 12.5/1) and apoptotic (ANX/PI, TMRE) and oxidative stress (MSOX, C-DCF and glutathione (GSH)) parameters were determined. ANX/PI staining is expressed as a percentage of positive cells in exposed hemocytes with respect to controls. TMRE, MSOX, C-DCF and GSH data are reported as Mean Fluorescence Intensities (MIF) with respect to controls. See methods for details. Data, representing the mean ± SD of 4 experiments, were analysed by ANOVA followed by Tukey’s post-hoc test. *p < 0.01 (treatment vs. control); #*p* < 0.05 (25 vs. 50 μg/ml).

The effects of hemocyte incubation with OlyA6/PlyB (25 and 50 μg/ml) on apoptotic and oxidative stress markers were quantified by flow cytometry and the results are reported in Figure 9. With regards to apoptosis-related parameters, both concentrations of the mixture induced a significant increase in the ratio Annexin V-FITC (ANX)/propidium iodide (PI) (+115 and +170%, respectively, vs. controls). Moreover, a significant increase in TMRE fluorescence was detected at 50 μg/ml (+44% with respect to controls). For oxidative stress parameters, MitoSOX and C-DCF fluorescence significantly increased at both concentrations (+25 and +115% for MitoSOX; +117 and +97%, for C-DCF, respectively, vs. controls), while a decrease in glutathione signal was detected (-35 and -39%, respectively, vs. controls) (Figure 9).

## 3 Discussion

Cellular membranes are composed of several thousands of chemically different lipids that serve many functions, such as structural membrane components, signalling molecules, or platforms for protein recruitment (Harayama and Riezman, 2018). Lipids control these biological processes by regulating membrane properties, and alterations in membrane lipid homeostasis is consequently associated with various diseases (Horn and Jaiswal, 2019; Nishimura and Matsumori, 2020). In contrast to proteins that can be prone to mutations, lipids are structurally conserved and constitutive components of cell membranes. This feature makes membrane lipids and lipid domains, like membrane rafts, ideal targets for treatment of various diseases (e.g., neurodegeneration, neuropatic pain, atherosclerosis, and infection, including the one with Sars-CoV-2) (D’Angelo et al., 2018; Resnik et al., 2015; Sviridrov et al., 2020a; Sviridrov et al., 2020b), and for the development of new biopesticides (Panevska et al., 2019a).

Sphingolipids, together with glycerophospholipids and sterols, are the most abundant components of cell membranes, and their sphingosine-based degradation products are involved in many physiological processes, including signalling events (Panevska et al., 2019b). In mammalian cell membranes, interactions between sphingomyelin and Chol drive the formation of membrane rafts; heterogeneous, dynamic membrane nanodomains that serve as functional platforms that regulate various cellular processes (e.g., immune signalling, host-pathogen interactions, development and regulation of cardiovascular disease and cancer) as a result of the segregation of specific lipid-anchored proteins within raft domains (Pike, 2006; Michel and Bakovic, 2007; Lingwood and Simons 2010; Sezgin et al., 2012). While mammalian cell membranes contain sphingomyelin as the major sphingolipid, invertebrates, especially arthropods, synthesize its analogue with a phosphoethanolamine instead of phosphocholine polar head, the CPE. Complete understanding of the physiological relevance of CPE and its biological role is still lacking, but recent experimental evidences suggest that this sphingolipid might be crucial for the early development of *Drosophila melanogaster*, where it has a key role in axonal ensheathment of peripheral nerves by glia (Ghosh et al., 2013), and it might be involved in the developmental stages of *Trypanosoma brucei* (Suttervala et al., 2008; Bhat et al., 2015). In contrast to sphingomyelin, the membrane behavior of CPE has been considerably less studied, but it was suggested that it does not induce the formation raft-like domains (Ramstedt and Slotte, 2006). The biological role of CAEP, a CPE analogue characteristic of marine invertebrates (Moschidis, 1984; Tomonaga et al., 2017; Imbs et al., 2019) bearing a phosphono rather than the usual phosphate group in its polar head, is even more obscure. Our lipidomic analyses of *M. galloprovincialis* hemocytes revealed the presence of 29.0 mol% CAEP among all the phospholipids, matching with the literature records of the whole mussel lipidome (Donato et al., 2018). This amount is considerably higher than the usual amount of CPE in insect cell membranes, where this lipid represents around 2–6 mol% (Guan et al., 2013) of total lipids.

Proteins from the aegerolysin family, such as OlyA6 from the edible oyster mushroom, were shown to specifically target sphingomyelin, that is the major sphingolipid in vertebrates, and CPE, that dominates in invertebrates (Sepčić et al., 2004; Bhat et al., 2015; Panevska et al., 2019a). Further, OlyA6/PlyB complexes can efficiently permeabilize membranes enriched in sphingomyelin or CPE, acting as potent and species-selective bioinsecticides (Panevska et al., 2019b). Because of a distinctive difference in the affinity to insect-specific sphingolipid CPE, which is their high-affinity receptor, and due to the fact that they are immediately degraded by mammalian digestive enzymes (Kristina Sepčić, personal communication), insecticides based on OlyA6/PlyB complexes are safe for vertebrates. However, this might not be the case with other organisms which contain CPE or its analogues in their cell membranes.

Within this study, we showed that a CPE analogue characteristic for marine invertebrates, the CAEP, also acts as an OlyA6 high-affinity receptor. OlyA6 was able to strongly bind to artificial lipid vesicles supplemented with CAEP isolated from mussel hemocytes, and OlyA6/PlyB mixture efficiently permeabilized CAEP-supplemented lipid vesicles and induced the apoptosis of *Mytilus* haemocytes at submicromolar concentrations. Short-term exposure of mussel hemocytes to the OlyA6/PlyB mixture affected the function of lysosomal membranes, as indicated by the concentration-dependent decrease in lysosomal membrane stability, with an EC_50_ of 6.5 μg/ml (corresponding to 0.43 μM OlyA6). In the 5–10 μg/ml range, a significant decrease in phagocytic activity was observed, indicating a general disturbance of membrane function, that is probably the consequence of the formation of OlyA6/PlyB transmembrane pores (Lukoyanova et al., 2015; Ota et al., 2013; Milijaš Jotić, 2021). Higher concentrations were ineffective; however, since the phagocytosis assay is based on microscopic observations of internalized NR-conjugated zymosan, the lack of effect in these conditions was due to non-specific, passive particle uptake by dying cells (see also Figure 6B). Moreover, phagocytic activity, as other functional immune parameters in bivalves, often shows a non-monotonic dose-response curve to chemical exposure (Balbi et al., 2021).

At increasing concentrations, progressive lysosomal destabilization was associated with cell rounding (indicating colloid-osmotic mechanism of cell disruption), detachment and death. At the highest concentration tested, only plasma membranes could be detected (“ghost” hemocytes), with complete loss of cytoplasm and organelles. To our knowledge, such an effect has not been previously observed in mussel hemocytes exposed in the same experimental conditions to a variety of potentially toxic inorganic and organic chemicals.

The results of both confocal laser scanning microscopy and flow cytometry provided a further insight into the toxic effects of OlyA6/PlyB on mussel hemocytes in the range of 10–50 μg/ml. Increases in parameters related to apoptotic processes both at the plasma membrane (Annexin V binding) and at mitochondrial level (TMRE) were observed. Moreover, OlyA6/PlyB induced increased oxyradical production both at mitochondrial (MitoSOX) and cytosolic (C-DCF) level, and provoked a decrease in reduced glutathione, indicating oxidative stress conditions. Deregulation of mitochondrial activity, leading to the cell death, has been already described in nucleated cells exposed to higher concentrations of bacterial pore-forming toxins (Kennedy et al., 2009; Bischovberger et al., 2012). It is thus very likely that the above-described effects observed in mussel hemocytes treated with OlyA6/PlyB complexes, also derive from the ability of the tested proteins to induce the formation of bi-component multimeric pores in hemocyte membranes.

Taken together, the results presented in this study indicate the possible application of fluorescently tagged aegerolysins, such as OlyA6, as molecular markers for studying the biology and distribution of CAEP in membranes of living cells that synthesize this membrane sphingolipid. These molecular probes could be also used for biophysical studies of CAEP or CPE membrane dynamics, both in living cells and in artificial lipid systems. Finally, due to their specific binding to membrane CAEP, aegerolysin-based cytolytic complexes, such as OlyA6/PlyB, could be considered as potential agents for selective elimination of organisms that harbour this sphingolipid in their membranes. Such examples are bivalves and other fouling marine invertebrates, but also some important plant pathogen oomycetes (Moschidis, 1984).

## 4 Materials and Methods

### 4.1 Materials

#### 4.1.1 Chemicals

All chemicals used in the present study were from Merck (United States) unless specified otherwise. Wool grease Chol, POPC, and CPE were from Avanti Polar Lipids (United States). Cholesterol and POPC lipids were stored at −20°C and dissolved in chloroform prior to use. CPE was dissolved in 1 ml chloroform/methanol (9/1, v/v).

#### 4.1.2 Proteins

The OlyA6 and Δ48PlyB (henceforth PlyB) recombinant proteins were produced as described previously (Ota et al., 2013; Panevska et al., 2019a) and stored in aliquots at −20°C prior to use. The lytic activity of the OlyA6/PlyB mixture (OlyA6/PlyB molar ratio, 12.5/1) on CPE-containing lipid vesicles prepared in artificial sea water (Lake Products Company LLC, United States) was tested prior to use and was found to be comparable to the activity on the same vesicles prepared in vesicle buffer (140 mM NaCl, 20 mM Tris, 1 mM EDTA, pH 7.4).

#### 4.1.3 Target Organisms

Mussels (*M. galloprovincialis* Lam.), were purchased in 2021 from an aquaculture farm in the Ligurian Sea (La Spezia, Italy) and acclimatized in static tanks containing aerated artificial sea water at pH 7.9–8.1, 36 ppt salinity (1 L/animal), 16 ± 1°C, for 3 days. In order to assess hemocyte functional parameters, for each sample hemolymph was extracted from the adductor muscle of 8–10 individuals and pooled. Hemocyte monolayers were prepared as previously described (Balbi et al., 2018, 2019).

### 4.2 Methods

#### 4.2.1 Lipid Extraction From *M. galloprovincialis* Hemocytes

Hemolymph was extracted from at least 150 animals and was immediately centrifuged at 800 × g for 10 min, at room temperature. The cell pellet obtained was resuspended in 700 μl of milliQ water in order to induce the cell lysis. Then, 3 ml of chloroform/methanol (2:1 v/v) were added to initiate the lipid extraction. The samples were placed on ice and sonicated with a Tip Sonicator (UP200S Hielscher Ultrasonic Technology, Germany) for 20 min, at 100 W, 50% on/off cycle. At the end of the sonication, the suspension was vortexed thoroughly and the sample was centrifuged at 6,000 × *g* for 10 min, at 4°C to separate the two phases. The lower phase was collected, and the inter-layer sediment and the aqueous phase were extracted again as described above. The lower phases, containing the lipids, were mixed together and dried by N_2_ flux to avoid oxidation. Dried samples were weighted and stored at −80°C. Lipids were dissolved in 600 µl deuterated methanol and analysed by NMR and HPLC-coupled LCMS.

#### 4.2.2 Lipidomic Analyses

##### 4.2.2.1 Nuclear Magnetic Resonance Analysis

^1^H-NMR (400 MHz) and ^31^P-NMR (162 MHz) spectra of the lipid extract dissolved in MeOH-d_4_ were recorded at 300 K on a nuclear magnetic resonance (NMR) spectrometer (400 MHz; Bruker-Avance, Bremen, Germany), with a 5-mm double resonance broadband observe probe with pulsed-gradient field utility. The 1H-90° proton pulse length was 9.3 µs, with transmission power of 0 db. The ^31^P-90° proton pulse length was 17 µs, with transmission power of −3 db. The probe temperature was maintained at 300.0 K (±0.1 K) using a variable temperature unit (B-VT 1000; Bruker). Calibration of the chemical shift scale (δ) was performed on the residual proton signal of the MeOH-d_4_ at δ_H_ 3.310 and δ_C_ 49.00 ppm, and the phosphatidylcholine (PC) signal at δ_P_ −0.550 ppm was used for calibration of the ^31^P-NMR δ scale. The following measurements were performed: ^1^H-NMR (i.e., proton chemical shifts, scalar couplings); ^31^P-NMR composite pulse decoupling to remove any proton coupling in ^31^P-NMR spectra, where generally 4,000 free induction decays were acquired and processed using exponential line broadening of 0.3 Hz prior to Fourier transformation. The resulting 1D-NMR spectra were analysed using TopSpin 3.6.1 (Bruker, Bremen, Germany). The lipid classes from the NMR data were identified through comparisons with our previous NMR measurements carried on commercially available lipid standards.

##### 4.2.2.2 HPLC-Electrospray Ionization-Mass Spectrometry Analysis

The lipid extract was analysed by liquid chromatography-mass spectrometry (LC-MS) (Model 1,100 series; Hewlett-Packard) coupled to a quadrupole ion-trap mass spectrometer (Esquire LCTM; Bruker, Bremen, Germany) equipped with an electrospray ionisation source and in both positive and negative ion modes. Chromatographic separation of the phospholipids was carried out at 303 K on a thermostated C18 column (Kinetex 2.6 µ; length, 100 mm; particle size, 2.6 µm; internal diameter, 2.1 mm; pore size, 100 Å; Phenomenex, Torrence, CA, United States). The solvent system consisted of eluant A as MeOH/H2O (7:3, v/v) containing 10 mM ammonium acetate and eluant B as isopropanol/MeOH (10:90, v/v) containing 10 mM ammonium acetate. Samples were resuspended in 1 ml CHCl_3_/MeOH (2:1, v/v), and 10 µl was run with a linear gradient from 65% eluant B to 100% B in 40 min, plus 20 min isocratic 100% B at 1 ml/min, to elute the diglycerides and triglycerides. The column was then re-equilibrated to 65% B for 10 min. The MS scan range was 13,000 U/s in the range of 50–1,500 m/z, with a mass accuracy of ∼100 ppm. The nebuliser gas was high purity nitrogen at a pressure of 20–30 psi, at a flow rate of 6 L/min and at 300°C. The electrospray ionisation was operated in positive ion mode for the qualitative and quantitative analyses of PC, lyso-PC, and sphingomyelin, and in both positive and negative ion modes for phosphatidylinositol, PE and CAEP. For the structural assignments of the lipid species, the extracted ion chromatograms from the positive and/or negative ion full scan data were integrated using the DataAnalysis 3.0 software (Bruker Daltonik, Bremen, Germany).

##### 4.2.2.3 CAEP Isolation

CAEP isolation was performed by HPLC (Agilent, model 1,100 series; Hewlett-Packard). Separation was achieved by isocratic gradient of 95% MeOH + ammonium acetate 10 mM and 5% ddH_2_O on a C18 column (Kinetex 2.6 µ; length, 100 mm; particle size, 2.6 µm; internal diameter, 2.1 mm; pore size, 100 Å; Phenomenex, Torrence, CA, United States) at flow 1 ml/min. UV chromatograms were acquired at 232 nm.

#### 4.2.3 Preparation of Lipid Vesicles

Equimolar multilamellar vesicles containing CEAP isolated from mussels’ hemocytes (CAEP/POP/Chol) or commercial CPE (CPE/POPC/Chol) were prepared (final concentration 5 mg/ml) in 140 mM NaCl, 20 mM Tris, 1 mM EDTA, pH 7.4, as described previously (Sepčić et al., 2003). To prepare the LUVs, suspensions of multilamellar vesicles were subjected to five freeze-thaw cycles and then extruded through 0.1 µm polycarbonate filters (Millipore, Germany) at ∼50°C. Equimolar small unilamellar vesicles CAEP/POPC/Chol and CPE/POPC/Chol loaded with calcein at the self-quenching concentration (80 mM) were prepared for the calcein release experiment as described previously (Sepčić et al., 2003).

#### 4.2.4 Surface Plasmon Resonance-Based Binding Studies

The interaction of OlyA6 and OlyA6/PlyB with CAEP/POPC/Chol (1/1/1, mol/mol/mol) and CPE/POPC/Chol (1/1/1, mol/mol/mol) LUVs was monitored with a surface plasmon resonance-based refractometer (Biacore T200; GE Healthcare, United States) using an L1 sensor chip with 20 mM Tris, 140 mM NaCl, 1 mM EDTA, pH 7.4, as running buffer. After initial cleaning of the chip with regeneration solutions of sodium dodecyl sulphate and octyl-β-D-glucopyranoside with 1-min injections at a flow rate of 10 μl/min, LUVs were bound to the second flow cell of the sensor chip to reach responses of ∼8,000 RU. The first flow cell was left empty to control for possible non-specific binding of the proteins to the dextran matrix of the chip. Non-specific binding of the proteins was minimized by a 1-min injection of 0.1 mg/ml bovine serum albumin at a flow rate of 30 μl/min. A single-cycle kinetics experiment was performed in which OlyA6 and OlyA6/PlyB (molar ratio 12.5/1) were injected at concentrations 0.03, 0.06, 0.12, 0.25, and 0.5 µM, with no dissociation in between and a dissociation time of 180 s at the end. Chip regeneration was achieved with 1-min injections of 0.5% sodium dodecyl sulphate and 40 mM β-D-glucopyranoside at a flow rate of 10 μl/min. Experiments were performed at 25°C. Data were processed using BIAevaluation software (GE Healthcare).

#### 4.2.5 Permeabilization of the Small Unilamellar Vesicles

Vesicle permeabilization was determined using a fluorescence microplate reader (Tecan, Switzerland), with excitation and emission set at 485 and 535 nm, respectively. Calcein-loaded CAEP/POPC/Chol (1/1/1, mol/mol/mol) and CPE/POPC/Chol (1/1/1, mol/mol/mol) small unilamellar vesicles were exposed to OlyA6/PlyB (molar ratio 12.5/1) at concentrations ranging from 0.0039 to 0.5 µM. Experiments with OlyA6 or PlyB alone were run in parallel. The experiments were performed for 20 min at 25°C. The permeabilization induced by the lytic OlyA6/PlyB complex was expressed as a percentage of the maximum permeabilization obtained by adding the detergent Triton-X 100 at a final concentration of 1 mM.

#### 4.2.6 Effect of Aegerolysin-Based Complexes on *Mytilus galloprovincialis* Hemocytes

##### 4.2.6.1 Hemocyte Functional Parameters

Lysosomal membrane stability was evaluated by the Neutral Red retention time (NRR) assay as in Balbi et al. (2018), (2019). Hemocyte monolayers were pre-incubated with different concentrations of OlyA6 (5, 10, 25, 50, and 500 μg/ml) in combination with PlyB, at an OlyA6/PlyB molar ratio of 12.5/1 for 30 min. Cells were then incubated with 20 μl of Neutral Red (Sigma-Aldrich, Milan, Italy) solution (final concentration 40 μg/ml from a stock solution of neutral red 40 mg/ml in DMSO). Experiments with OlyA6 (25 μg/ml) or PlyB alone (2 μg/ml) were run in parallel. All incubations were performed at 16°C. After 15 min, excess of dye was washed out, 20 μl of artificial sea water was added, and slides were sealed with a coverslip. Every 15 min, slides were examined using an inverted Olympus IX53 microscope (Olympus, Milano, Italy), equipped with a CCD UC30 camera and a digital image acquisition software (cellSens Entry). The endpoint of the assay was defined as the time at which 50% of the cells showed signs of lysosomal leaking (the cytosol becoming red and the cells rounded). For each experiment, control hemocyte samples were run in parallel. Triplicate preparations were made for each sample. Data are expressed as % of control.

Phagocytosis of neutral red-stained zymosan by hemocyte monolayers was used to assess the phagocytic ability of hemocytes. Neutral red-stained zymosan in 0.05 M Tris-HCl buffer (TBS), pH 7.8, containing 2% NaCl was added to each monolayer at a concentration of about 1:30 hemocytes:zymosan in the presence or absence of OlyA6/PlyB mixture (5, 10, 25, and 50 μg/ml), and allowed to incubate for 60 min. Monolayers were then washed three times with TBS, fixed with Baker’s formol calcium (4%, v/v, formaldehyde, 2% NaCl, 1% calcium acetate) for 30 min and mounted in Kaiser’s medium for microscopical examination. For each slide, the percentage of phagocytic hemocytes was calculated from a minimum of 200 cells. All experiments were performed in quadruplicate samples (N = 4).

##### 4.2.6.2 Confocal Laser Scanning Microscopy

For confocal live imaging, aliquots of hemolymph (500 μl) exposed to OlyA6/PlyB mixture (10 and 25 μg/ml, at an OlyA6/PlyB molar ratio of 12.5/1), were seeded for 30 min in glass bottom culture dish (MatTek, Ashland, MA) and stained with 1) tetramethylrhodamine ethyl ester (TMRE) (40 nM for 10 min), for determination mitochondrial membrane potential; 2) the mitochondrial reactive oxygen species-sensitive probe MitoSOX Red (5 μM, 10 min), and 3) 5-(and-6)-chloromethyl-20,70-dichlorodihydrofluorescein diacetate acetyl ester, CM-H2DCFDA (2 µM for 20 min), for generation of intracellular H_2_O_2_. All probes were diluted in artificial sea water. All reagents were from Molecular Probes Inc.

Fluorescence of TMRE (ex: 568 nm, em: 590–630 nm), MitoSOX Red (ex: 488 nm, em: 580 nm) and DCF (ex: 495 nm, em: 520 nm) were detected using a Leica TCS SP5 confocal setup mounted on a Leica DMI 6000 CS inverted microscope (Leica Microsystems, Heidelberg, Germany) using 63 ×1.4 oil objective (HCX PL APO ×63.0 1.40 OIL UV). Images were analysed by the Leica Application Suite Advanced Fluorescence (LASAF) and ImageJ Software (Wayne Rasband, Bethesda, MA).

##### 4.2.6.3 Flow Cytometry

Aliquots (200 μl) of whole hemolymph (each containing about 1–2 × 10^6^ cells/ml) were incubated with the OlyA6/PlyB mixture (25 and 50 μg/ml) for 30 min at 16°C. Control samples were run in parallel. Total hemocyte count was carried out using an Omnicyt flow cytometer (Cytognos SL, Salamanca, Spain). Samples were pelleted by centrifugation (100 × g for 10 min) and stained with different fluorophores. Annexin V-FITC (ANX)/propidium iodide (PI) and TMRE staining was carried out as previously described (Ciacci et al., 2012; Canesi et al., 2015); MitoSOX and CM-H2DCFDA staining was performed as described above for confocal scanning laser microscopy. Samples were also analysed by the Intracellular Glutathione Detection Assay Kit (Abcam) (at ex: 490, em: 520 nm). Sample acquisition and analyses were performed by means of a FACS Canto II flow cytometer and analysed with DiVa™ software collecting at least 10,000 events for each sample. Data, representing the mean ± standard deviation (SD) of at least three experiments, are expressed as Mean Fluorescence Intensities reported as percent changes with respect to controls, except for ANX/PI staining, where data are expressed as percent positive cells with respect to controls.

##### 4.2.6.4 Statistics

Data are the mean ± SD of four independent experiments. Statistical analysis was performed using the non-parametric Mann-Whitney *U* test or ANOVA followed by Tukey’s post hoc test. The EC_50_ for lysosomal membrane stability was calculated from a regression model of the original data and analysed by one-way ANOVA at a 95% confidence interval. All statistic calculations were performed by the PRISM seven GraphPad software.

## Data Availability

The original contributions presented in the study are included in the article/Supplementary Material, further inquiries can be directed to the corresponding authors.
