# Supplementary material for: Ceramide Aminoethylphosphonate as a New Molecular Target for Pore-Forming Aegerolysin-Based Protein Complexes
Source: Front Mol Biosci. 2022 May 25;9:902706. doi: 10.3389/fmolb.2022.902706 (PMC9174665; doi:10.3389/fmolb.2022.902706)
Supplement: Supplementary file 1 [file DataSheet1.PDF]

Supplementary Material

Ceramide aminoethylphosphonate as a new molecular target for pore-forming aegerolysin-based protein complexes

Teresa Balbi[1,†], Francesco Trenti[2, †], Anastasija Panevska[3], Gregor Bajc[3], Graziano Guella[2,], Caterina Ciacci[4], Barbara Canonico[4], Laura Canesi[1,*], Kristina Sepčić[3,*]

[1] Department of Earth, Environmental and Life Sciences, University of Genoa, Genoa, Italy

[2] Bioorganic Chemistry Laboratory, Department of Physics, University of Trento, Trento, Italy

[3] Department of Biology, Biotechnical Faculty, University of Ljubljana, Ljubljana, Slovenia

[4] Department of Biomolecular Sciences, University of Urbino Carlo Bo, Urbino, Italy

***Correspondence:**

Kristina Sepčić

kristina.sepcic@bf.uni-lj.si

Laura Canesi

laura.canesi@unige.it

[†] These authors have contributed equally to this work.

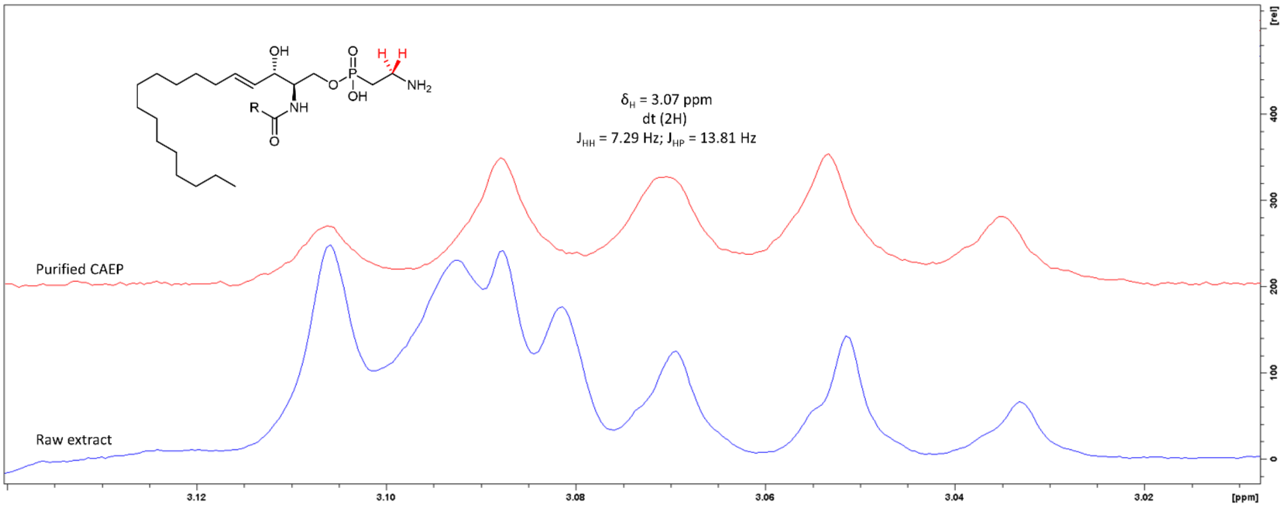

Supplementary Figure 1: Diagnostic 1H-NMR signal of CAEP phosphonate head. In purified CAEP (red, upper spectrum) the highlighted protons appear as a doublet of triplets. In the raw extract (blue, lower spectrum) the CAEP diagnostic are overlapping with the corresponding PE protons.

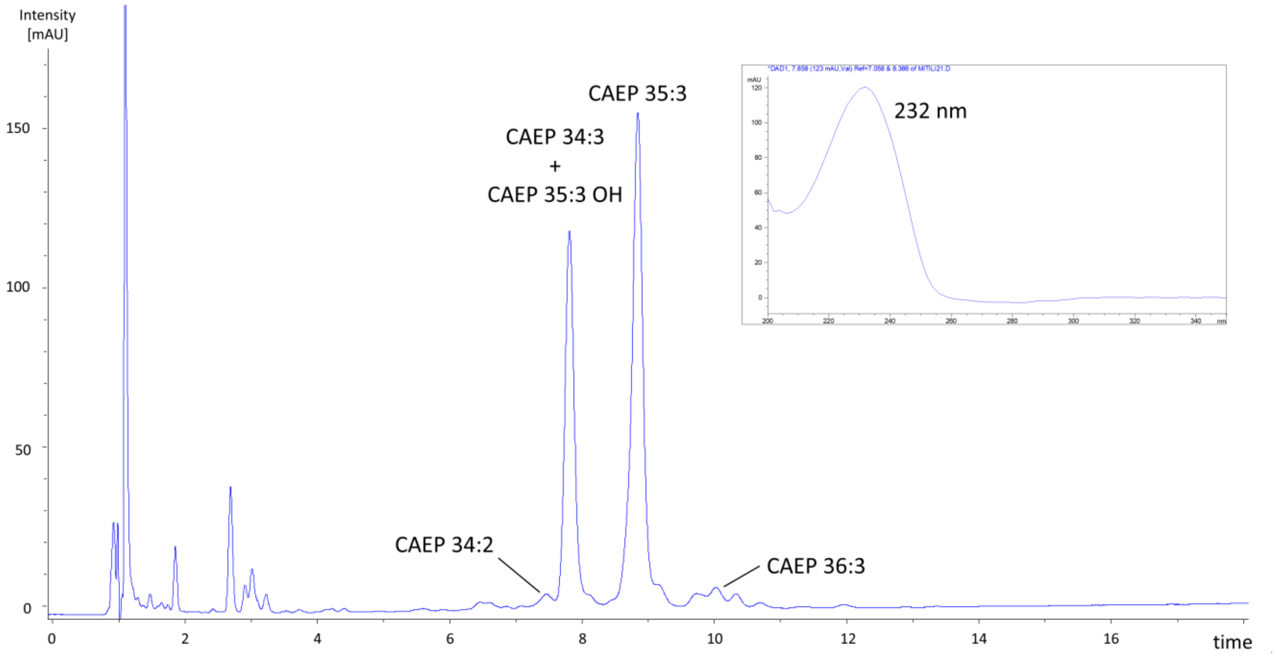

Supplementary Figure 2: HPLC chromatograms of *M. galloprovincialis* raw lipidome acquired at 232 nm. The two major peaks and adjacent regions (range between 7 to 11 minutes) were isolated

yielding a mixture of five co-eluting major CAEP plus minor ones (<3 mol%). Note that CAEP 34:2 and 36:3 did not absorb at 232 nm, but were nevertheless collected into the CAEP mix.
